# Should Injectable Meloxicam Be Approved for Use in Cats?

**DOI:** 10.1111/jvp.70060

**Published:** 2026-03-08

**Authors:** Matthew K. Wun, Michael H. Court, Nicolas F. Villarino, Richard Malik

**Affiliations:** ^1^ Department of Veterinary Clinical Sciences, College of Veterinary Medicine Washington State University Pullman Washington USA; ^2^ Department of Pharmacology and Toxicology, College of Pharmacy University of Arizona Tucson Arizona USA; ^3^ VetMED Emergency & Specialty Veterinary Hospital Phoenix Arizona USA; ^4^ Centre for Veterinary Education The University of Sydney Camperdown New South Wales Australia

**Keywords:** acute kidney injury, adverse drug reactions, NSAID, research integrity

## Abstract

Injectable meloxicam remains widely used in feline practice despite its association with acute kidney injury (AKI). Evidence of this association was apparent in the results of a clinical trial submitted to the U.S. Food and Drug Administration as part of a New Animal Drug Application (NADA) Freedom of Information Summary. These data, however, were omitted from the published version of that clinical trial in the Journal of the American Veterinary Medical Association (JAVMA). The study's financial sponsorship by the drug manufacturer raises ethical concerns, especially given meloxicam's ongoing and enthusiastic promotion by veterinary organizations also financially supported by the manufacturer. This viewpoint questions meloxicam's approval process and highlights the potential influence of pharmaceutical industry sponsorship on veterinary prescribing practices and policy.

Although widely prescribed by veterinarians, injectable NSAIDs have been associated with acute kidney injury (AKI), particularly in cats (Wun et al. 2025). Most contemporary reports in cats have involved the original formulation of meloxicam (Metacam, Boehringer Ingelheim) (Wun et al. 2023; Krekis et al. 2024). It is worth examining how this drug came to be approved in the U.S., the rationale behind it, and why injectable NSAIDs have become so ingrained into routine feline anesthesia and analgesia protocols (Grubb et al. 2020; Steagall et al. 2022).

Meloxicam 5 mg/mL solution for injection was approved for use in cats by the U.S. Food and Drug Administration (FDA) in 2004. Prior to that, meloxicam was available as an oral formulation for use in dogs, and this formulation was often used off‐label for cats at a dose of 0.05 mg/kg/day. As part of the New Animal Drug Application (NADA) Freedom of Information Summary, the results from a clinical trial evaluating the effectiveness and safety of meloxicam for the control of postoperative pain and inflammation associated with onychectomy, with and without concurrent neutering, compared to butorphanol, were reported (U.S. Food and Drug Administration 2004). In that study, cats were administered either butorphanol (0.4 mg/kg via subcutaneous (SC) injection) or meloxicam (0.3 mg/kg via SC injection) once preoperatively, and serum biochemistry values were recorded at baseline and 24 h after surgery. Of the cats that received meloxicam, 6/72 cats (8.3%) developed an elevated serum BUN concentration (outside of the reference interval) post‐treatment, compared to none of the 66 cats that received butorphanol. Three of these six cats (3/72; 4.2%) in the meloxicam group also developed a concurrent increase in serum creatinine (U.S. Food and Drug Administration 2004). Although serum creatinine values were reported to be within an undefined ‘normal range’, IRIS grade I AKI is possible if the serum creatinine concentration increased by ≥ 0.3 mg/dL (Cowgill 2016).

It is not clear why, but these findings were omitted in the version of this study published in the Journal of the American Veterinary Medical Association JAVMA! (Carroll et al. 2005). The authors concluded that ‘Meloxicam appeared to have a good safety profile in the cats in our study’, and that “Preoperative administration of meloxicam did not result in significant differences in platelet count or concentrations of creatinine, sodium, potassium, chloride, PCV, BUN, or hemoglobin, compared with butorphanol.” While the total range (i.e., lowest value to highest value) of the serum biochemistry values were provided in the NADA (in which the elevated BUN seen in some cats became evident), neither the range or the BUN or creatinine concentrations for individual cats were reported in the JAVMA article in the text or in a graphical format; only the means and standard deviations of the values were provided (Table 6 in Carroll et al. 2005). By selecting standard deviation as their measure of dispersion (which was statistically inappropriate given that the data was not determined to be normally distributed), the authors have effectively ‘buried’ the fact that six cats developed elevated serum BUN concentrations following SC meloxicam use. It would have been more transparent to provide these data as scatter plots or stem and whisker plots, as in this experiment, critically, it's the outliers that are actually the most important individuals to be reported when evaluating drug safety under field conditions. We have requested this information from the corresponding author of this article via email, who responded only that she had retired (personal communication). We believe that this is a substantial research integrity issue, especially considering that the third author of this study was affiliated with the drug company that manufactured the drug used in this trial, and because this article is frequently cited to provide evidence of the safety of injectable meloxicam (Sparkes et al. 2010; Lascelles et al. 2007).

If all three of the cats in the study that received SC meloxicam (0.3 mg/kg) did, in fact, develop an IRIS grade I AKI, this would equate to an approximate 4% frequency of this adverse effect. What we do not know is what percentage of a large population of cats would develop more severe AKI if given meloxicam under the same circumstances. Given no evidence for the necessity or superiority over alternative analgesic drugs (Wun et al. 2025), we question whether injectable meloxicam meets the FDA's requirement that a drug's expected benefits outweigh its potential risks to patients (U.S. Food and Drug Administration 2018). Furthermore, given the mounting evidence for risk (Wun et al. 2025), we are curious why the use of this drug in cats has not attracted more criticism and skepticism. Could Boehringer Ingelheim's sponsorship of key opinion leaders, including International Cat Care and the WSAVA Global Pain Council, be a contributing factor? (World Small Animal Veterinary Association 2024; International Cat Care 2024). In Australia, Boehringer Ingelheim and the Australian and New Zealand College of Veterinary Scientists (ANZCVS) Veterinary Anaesthesia and Analgesia Chapter have collaborated to host an online pain management continuing education module (Boehringer Ingelheim Animal Health New Zealand Ltd. 2024) and, in 2023, published a technical bulletin together titled ‘Clinical recommendations for peri‐operative use of meloxicam injection in cats’, which was disseminated through the Australian Veterinary Practitioners Board of New South Wales website (ANZCVS Veterinary Anaesthesia & Analgesia Chapter 2023). This perception of a close relationship between the ANZCVS, the New South Wales Veterinary Practitioners Board, and Boehringer Ingelheim is, in our view, unsettling and deserves explanation. Upon questioning, the Chief Executive Officer of the ANZCVS refused to comment on whether the College and its Chapters have received funding from Boehringer Ingelheim (personal correspondence).

It is well‐documented in human medicine that donations from pharmaceutical companies to professional medical societies can influence prescribing behavior and policy (Lin et al. 2017; MacKenzie et al. 2023), and that clinical trials funded by pharmaceutical companies are strongly associated with results that favor the sponsors' interests (Sismondo 2008). Taking this meloxicam ‘story’ as an example, we believe our profession must be equally vigilant.

## Author Contributions

M.K.W.: conception, literature review and analysis, and manuscript drafting and revision; M.H.C.: literature review and analysis, and manuscript revision; N.F.V.: literature analysis and manuscript revision; R.M.: conception, literature review and analysis, and manuscript drafting and revision.

## Funding

The authors have nothing to report.

## Disclosure

The authors have nothing to report.

## Conflicts of Interest

The authors declare no conflicts of interest.

## Data Availability

Data sharing not applicable to this article as no datasets were generated or analyzed during the current study.
